# Development of Fluorescent Sensors for Biorelevant Anions in Aqueous Media Using Positively Charged Quantum Dots

**DOI:** 10.3390/mi15030373

**Published:** 2024-03-09

**Authors:** Hitalo J. B. Silva, Claudete F. Pereira, Goreti Pereira, Giovannia A. L. Pereira

**Affiliations:** 1 Departamento de Química Fundamental, Universidade Federal de Pernambuco, Recife 50740-560, PE, Brazil; hitalo.silva@ufpe.br (H.J.B.S.); claudete.fernandes@ufpe.br (C.F.P.); 2 Departamento de Química & CESAM, Universidade de Aveiro, 3810-193 Aveiro, Portugal

**Keywords:** semiconductor nanocrystals, detection, bicarbonate, carbonate, sulfate, bisulfate, water monitoring

## Abstract

Quantum dots (QDs) have captured the attention of the scientific community due to their unique optical and electronic properties, leading to extensive research for different applications. They have also been employed as sensors for ionic species owing to their sensing properties. Detecting anionic species in an aqueous medium is a challenge because the polar nature of water weakens the interactions between sensors and ions. The anions bicarbonate (HCO_3_^−^), carbonate (CO_3_^2−^), sulfate (SO_4_^2−^), and bisulfate (HSO_4_^−^) play a crucial role in various physiological, environmental, and industrial processes, influencing the regulation of biological fluids, ocean acidification, and corrosion processes. Therefore, it is necessary to develop approaches capable of detecting these anions with high sensitivity. This study utilized CdTe QDs stabilized with cysteamine (CdTe-CYA) as a fluorescent sensor for these anions. The QDs exhibited favorable optical properties and high photostability. The results revealed a gradual increase in the QDs’ emission intensity with successive anion additions, indicating the sensitivity of CdTe-CYA to the anions. The sensor also exhibited selectivity toward the target ions, with good limits of detection (LODs) and quantification (LOQs). Thus, CdTe-CYA QDs show potential as fluorescent sensors for monitoring the target anions in water sources.

## 1. Introduction

Sensing methodologies have been advancing with the evolution of scientific knowledge. Nevertheless, there is still a substantial interest in analytical detection systems with rapid responses, high precision, excellent sensitivity, and economical manufacturing. In this context, optical fluorescent sensors have emerged as promising sensing platforms, mainly the ones based on nanomaterials [1,2]. In general, nanoparticles enhance the sensor’s surface area, facilitating more effective interaction with the analyte and improving detection sensitivity [3,4,5].

Among the fluorescent nanomaterials, quantum dots (QDs) have gained prominence in sensor development. These fluorescent semiconductor nanocrystals, ranging from 1 to 10 nm, exhibit a broad absorption spectrum, a narrow emission band, and exceptional photostability. Notably, QDs possess a chemically active surface, facilitating their functionalization and enhancing their affinity with the analyte [6]. QDs have emerged as luminescent probes in analytical chemistry to develop various sensor types, including optical, electrochemical, and colorimetric [2,7,8].

The presence of ligands on the surface plays a critical role in conferring selectivity to nanosensors. These ligands are essential for stabilizing quantum dots (QDs) and significantly impacting their optical properties [9,10]. Additionally, the surface functionalization of QDs with specific ligands is crucial for imparting selectivity to the nanosensor, increasing the affinity between the probe and the analyte, and further enhancing the sensitivity and selectivity of the sensor [11]. Typically, ligands feature a thiol group attached to the nanoparticle surface and a terminal group such as carboxylic acid (-COOH) or amine (-NH_2_). The charge carried by these groups depends on the surrounding pH with electrostatic phenomena regulating interactions between QD surface ligands and ions, as well as hydrogen bonds or van der Waals forces [12,13].

For this reason, regarding studies in aqueous media, controlling the pH of the reaction medium is crucial as it directly influences the ionization of the stabilizer functional groups. pH values higher or lower than the acid dissociation constants (pKa) of these groups dictate their protonation state, affecting electrostatic interactions with species in the medium. Consequently, the acid–base equilibrium is dynamic and reliant on the chemical environment and pH conditions of the reaction medium [14].

On the other hand, the phenomenon of fluorescence has been widely employed as the main detection mechanism in optical sensors. In the presence of the analyte, the emission intensity of quantum dots (QDs) may undergo either an increase or suppression, depending on the specific interaction between the nanosensor and the analyte [15]. Notably, studies exhibiting emission intensity suppression have been more prevalent in the literature, mainly due to the fluorescence resonance energy transfer (FRET) phenomenon. However, approaches showing an enhancement in the fluorescence can also occur and usually offer more challenges to comprehend and explain [15,16].

The current literature on ion detection in aqueous media predominantly focuses on metallic cations, taking advantage of the coordination bonding between some organic molecules and metals [17,18]. Although the detection of anionic species in organic media has been explored, investigations in aqueous environments remain limited. Challenges persist in aqueous detection due to water’s polar nature, which weakens interactions between recognition substances and anions. Recent sensor developments for anionic species in aqueous media are based on macrocycles, such as calix[n]arenes, polyamides, cyclodextrins, and urea derivatives [19,20,21].

The chalcogenide QDs developed to detect anions in an aqueous medium required the surface modification of the nanocrystals with polymers, metal complexes, or other nanoparticles, introducing additional complexity to the sensor preparation and the sensing mechanism. For example, Pengpumkiat et al. [22] developed a fluorescent sensor for CN^−^ using CdTe QDs coated with chitosan and Cu^2+^ ions. In the presence of the copper ions, the QDs’ emission was quenched, and it was restored upon the addition of CN^−^. To detect fluoride, Zhang et al. [23] prepared silica nanospheres containing two QDs (a green- and a red-emitting QD) and mixed them with 2-(tert-butyldiphenylsilyloxy)phenol (2-TBDPSP). According to these authors, the addition of F^−^ promoted the Si–O bond cleavage, releasing a quinone derivative that was then linked to the QD-silica nanospheres, provoking a decrease in the fluorescence signal. In another example, Jindal and Kaur [24] coated ZnO QDs with a benzimidazole derivative, synthesized by them in an organic medium, to detect bisulfide anions.

Monitoring some anions is crucial due to their abundance and significant roles in aquatic environments. Imbalances in ionic composition present severe consequences for the environment and ecosystems [19,20,21]. However, highly sensitive and selective fluorescent sensors designed specifically for the recognition of bicarbonate (HCO_3_^−^), carbonate (CO_3_^2−^), sulfate (SO_4_^2−^), and bisulfate (HSO_4_^−^) ions remain scarce. In freshwater, CO_3_^2−^ and HCO_3_^−^ ions, along with CO_2_, are the predominant carbonate compounds arising from various sources. In oceans, these anions play a vital role in regulating CO_2_ balance and preventing marine acidification, mitigating adverse effects from CO_2_ absorption [25,26,27].

Similarly, detecting anions such as HSO_4_^−^ and SO_4_^2−^ is essential, given their broad implications in both industrial and environmental contexts. HSO_4_^−^ is present in nuclear fuel, industrial waste, and agricultural fertilizers, with severe environmental impacts. Sulfate ions result from SO_2_ emissions into the atmosphere due to the combustion of fossil fuels, leading to concrete corrosion and water contamination. Thus, a fast and sensitive detection of sulfate ions is crucial for environmental monitoring, particularly in saline waste with high sulfate content, where excessive sulfate can promote the growth of sulfate-reducing bacteria, producing toxic sulfite [28,29,30].

In this work, we evaluated the application of positively charged QDs as sensors for anions in aqueous media. Specifically, QDs functionalized with cysteamine were prepared and utilized to develop analytical nanoplatforms for anion detection.

## 2. Materials and Methods

### 2.1. Materials

All analytical-grade materials were used as received without any further purification, and all solutions were prepared with ultrapure water (resistivity of 18.2 MΩcm at room temperature): cadmium chloride (CdCl_2_, 99.99%, Sigma-Aldrich, St. Louis, MO, USA), sodium tellurite (Na_2_TeO_3_, 99%, Sigma-Aldrich), sodium borohydride (NaBH_4_, 99.99%, Sigma-Aldrich), sodium hydroxide (NaOH, 98%, Sigma-Aldrich), cysteamine hydrochloride (CYA, 98.0%, Sigma-Aldrich), 3-mercaptosuccinic acid (MSA, 97%, Sigma-Aldrich), L-glutathione reduced (GSH, 98.0%, Sigma-Aldrich), anhydrous sodium carbonate (Na_2_CO_3_, 99.7%, NEON, Suzano, SP, Brazil), sodium bicarbonate (NaHCO_3_, 100.0%, NEON), potassium sulfate (K_2_SO_4_, 99.0%, Química Moderna, Barueri, SP, Brazil), potassium bisulfate (KHSO_4_, P.A., Vetec, Duque de Caxias, RJ, Brazil), Sodium chloride (NaCl, 99.0%, Química Moderna), sodium nitrate (NaNO_3_, 99.0%, Vetec), potassium chloride (KCl, 99.0%, Vetec), potassium bromide (KBr, 99.0%, Dinâmica, Indaiatuba, SP, Brazil), potassium iodide (KI, 99.0%, Vetec), sodium acetate (CH_3_COONa, 99.0%, Vetec), disodium hydrogen phosphate dihydrate (Na_2_HPO_4_·2H_2_O, 99.0%, NEON, Suzano, SP, Brazil), monopotassium phosphate (KH_2_PO_4_, 99.0%, NEON, Suzano, SP, Brazil).

### 2.2. Preparation of CdTe Quantum Dots

The synthesis protocol employed in this study was based on the one-pot preparation method described by Viegas et al. (2019) [31], with modifications. To prepare CdTe quantum dots stabilized with cysteamine (CdTe-CYA), the synthesis was carried out with a fixed Cd:Te:CYA molar ratio of 10:1:12 [32]. Initially, 0.862 g (4.7 mmol) of CdCl_2_ was weighed and dissolved in 125 mL of ultrapure water in a two-neck round-bottom flask. Subsequently, 0.648 g (5.7 mmol) of cysteamine hydrochloride was added under magnetic stirring, and the pH of the solution was adjusted to 5.8 using a 2 M NaOH solution. The mixture was heated at 90 °C under a nitrogen atmosphere with constant magnetic stirring for 30 min. Then, a solution containing 0.0946 g (2.5 mmol) of NaBH_4_ in 1 mL of ultrapure water was prepared and injected into the reaction flask using a syringe, followed by the addition of 25 mL of a Na_2_TeO_3_ solution (0.02 M). The closed system was then heated at 90 °C and stirred continuously for 5 h, under an inert atmosphere. To prevent QD precipitation, the system pH was readjusted to 5.8 by adding additional cysteamine. After cooling, the quantum dots were stored under refrigeration.

CdTe QDs stabilized with MSA and GSH were prepared using a similar procedure, and using the Cd/Te/stabilizer molar ratio of 2:1:2.4.

### 2.3. Optical Characterization

The QD optical features were evaluated by UV–Vis absorption spectroscopy (Evaluation 600 Spectrophotometer, Thermo Scientific, Waltham, MA, USA) and emission spectroscopy (FluoroMax Plus, Horiba Scientific, Piscataway, NJ, USA). The sample was diluted in a QD/water ratio of 3:100 (*v*/*v*), and absorption and emission measurements were acquired with excitation at 405 nm. Consequently, parameters such as average particle size (nm), concentration (μmol·L**^−^**^1^) estimated, and full width at half maximum (FWHM) were determined. The average size or diameter of the nanoparticles, correlated with the first absorption peak, was calculated using the equation proposed by Dagtepe et al. (2007) [33] (Equation (1)). This methodological approach provides a systematic means of quantifying nanoparticle dimensions based on a spectral analysis.

(1) $$r=\frac{1.38435-0.00066\lambda }{1-0.00121\lambda }$$

where *r* is the average diameter of the nanoparticles and *λ* corresponds to the wavelength of the first maximum of the absorption spectrum.

The estimation of the extinction coefficient (ε) was calculated using the approximations proposed by Yu et al. (2003) [34] (Equation (2)), and the molar concentration of CdTe quantum dots (QDs) in suspension was estimated through Lambert–Beer’s law (Equation (3)).

(2) $$\varepsilon =10,043{\left(r\right)}^{2.12}$$

(3) A=εCl

where A is the absorbance corresponding to the absorption maximum, ε is the molar extinction coefficient, C is the molar concentration of the sample (mol·L**^−^**^1^), and l is the optical path length (cm) through which the radiation beam will pass for recording the absorption spectrum.

### 2.4. Evaluation of the Optical Response of CdTe-CYA QDs in the Presence of Different Anions

The optical properties (absorption and emission) of CdTe-CYA QDs were evaluated in the presence of specific anions (CO_3_^2−^, HCO_3_^−^, SO_4_^2−^, and HSO_4_^−^). Briefly, stock solutions of Na_2_CO_3_, NaHCO_3_, K_2_SO_4_, and KHSO_4_ salts were prepared at a concentration of 10 mmol·L**^−^**^1^. Subsequently, 60 µL of quantum dots was added to a quartz cuvette of a 10 mm path length, and the volume was adjusted to 2 mL with ultrapure water. Finally, the anionic analyte was gradually introduced into the suspension, ensuring a controlled addition process to observe the sensor’s response accurately. After each addition, the system underwent manual homogenization for a few seconds, guaranteeing thorough mixing and a consistent distribution of the analyte within the suspension. Following this step, absorption and emission spectra were acquired at room temperature, providing detailed insights into the interaction between the analyte and the CdTe-CYA QDs. To account for any effects of dilution, corrections were applied to the acquired spectra. This meticulous procedure was repeated three times to ensure the reliability and reproducibility of the obtained results.

### 2.5. Determination of Detection Parameters

From the titration-acquired data, calibration curves were generated for each anion. These curves were established by systematically analyzing the spectral profiles of the developed optical nanoprobes, using different known concentrations of anions to construct the response curves. Considering a univariate model, analytical curves were plotted to evaluate the linear range of the (F − F_0_)/F_0_ as a function of the analyte concentration (μM), where F_0_ and F are the fluorescence intensities in the absence and presence of the analyte, respectively. These studies were performed in triplicate. Calibration curves were statistically validated through an analysis of variance (ANOVA) at a 95% confidence level. This statistical analysis examined the data points for consistency and accuracy across the entire calibration range, aiming to provide reliability in measurements and ensure confidence in the sensor’s performance.

For analytical method validation, the limits of detection (LODs) and quantification (LOQs) were estimated based on the criteria established by the International Union of Pure and Applied Chemistry (IUPAC) [24]. This involved a systematic approach to assess the sensitivity and reliability of the method by establishing thresholds for the minimum detectable and quantifiable levels of analytes. By adhering to internationally recognized standards set by IUPAC, the validation process aimed to ensure robustness and accuracy in analytical measurements. Thus, LODs and LOQs were calculated using Equations (4) and (5):

(4) $$LOD=\frac{3\sigma }{\underset{.}{k}}$$

(5) $$LOQ=\frac{10\sigma }{\underset{.}{k}}$$

where *σ* corresponds to the standard deviation of the intercept, and *k* is the slope of the fitted curve. The linearity of the calibration curves was assessed using their respective determination coefficients (R^2^).

### 2.6. Selectivity

The sensor’s selectivity for target anions was assessed by testing solutions of various anions (10 mmol·L**^−^**^1^, including HPO_4_^2−^, H_2_PO_4_^−^, Cl^−^, I^−^, Br^−^, NO_3_^−^, SO_3_^−^, and CH_3_COO^−^) under identical controlled conditions. The anion solutions (243.9 µmol·L**^−^**^1^) were added to the diluted suspension of CdTe-CYA QDs, and after brief mixing, emission spectra were recorded at room temperature to analyze the response of the sensor to each specific anion. This systematic approach allowed for a comprehensive evaluation of the sensor’s selectivity and its capability to distinguish between different anions in a solution.

## 3. Results and Discussion

### 3.1. Characterization of CdTe-CYA QDs

**Figure 1:**
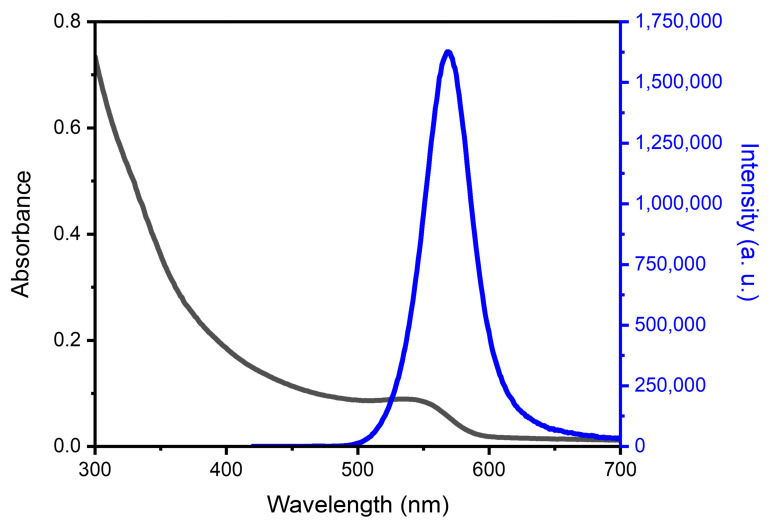
Absorption (black) and emission (blue) spectra of CdTe-CYA QDs (λ_exc_ = 405 nm).

According to the optical parameters of the CdTe-CYA QDs (Figure 1), we can observe that the nanomaterial exhibits a small Stokes shift, as well as an intense emission band, presenting an average diameter of 3.0 nm and a concentration of 28.9 mmol·L^−1^. In addition, other studies have reported the preparation of CdTe-CYA QDs with FWHM values ranging from 30 to 85 nm and sizes around 3.0 nm, consistent with the results obtained in the current study [32,35].

### 3.2. Fluorescent Detection of Anions Using CdTe-CYA QDs

To evaluate the sensing ability of CdTe-CYA QDs toward the target anions (CO_3_^2−^, HCO_3_^−^, SO_4_^2−^, and HSO_4_^−^), increasing concentrations of the analyte were added to the QDs, and absorbance and emission spectra were acquired. Firstly, this study was conducted by absorption spectroscopy to assess whether the core of the QDs would be affected by the presence of the target anions. It was observed that the position or width of the absorption spectra showed little or no variation, and there was no significant change in intensity, suggesting that the addition of anions did not cause changes in the core of the QDs, maintaining their composition and mean size (Figure S1).

**Figure 2:**
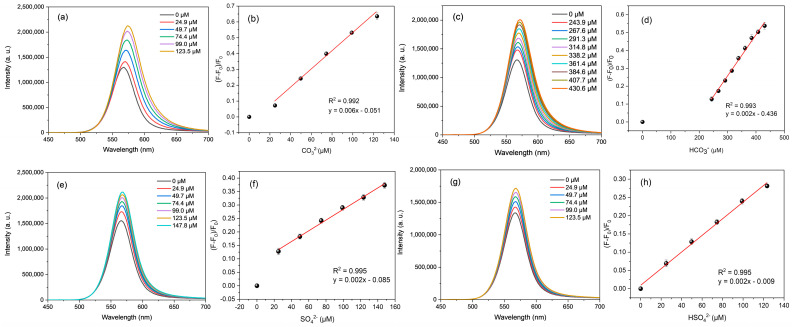
Fluorescence spectra of CdTe-CYA QDs (λ_exc_ = 405 nm) with different concentrations of CO_3_^2−^ (**a**), HCO_3_^−^ (**c**), SO_4_^2−^ (**e**), and HSO_4_^−^ (**g**), and calibration curves of (F − F_0_)/F_0_ versus analyte concentration: CO_3_^2−^ (**b**), HCO_3_^−^ (**d**), SO_4_^2−^ (**f**), and HSO_4_^−^ (**h**) concentration.

On the other hand, the addition of these anions to CdTe-CYA caused significant changes in the fluorescence profile (Figure 2). From the emission spectra of all systems, a consistent signal enhancement could be observed with increasing analyte concentrations.

The analysis performed allowed the estimation of the analytical parameters for the four sensor/analyte systems: CdTe-CYA/CO_3_^2−^, CdTe-CYA/HCO_3_^−^, CdTe-CYA/SO_4_^2−^, and CdTe-CYA/HSO_4_^−^.

As observed in Figure 2, the optical sensor exhibited a good correlation between the added CO_3_^2−^ concentration and the corresponding emission intensity, with a linear range between 43.1 and 123.5 μM (Figure 2b), a LOD and LOQ of 12.9 and 43.1 μM, respectively. For this anion, with a further addition of CO_3_^2−^, the emission intensity reached a plateau of around 291 μM, remaining relatively constant after that concentration. For the HCO_3_^−^ anion, the fluorescent sensor presented a linear range around higher values, between 107.33 and 430.62 μM (Figure 2d), without reaching a fluorescence plateau in this concentration range. Nevertheless, the LOD and the LOQ found were 32.20 μM and 107.33 μM, respectively. Regarding the SO_3_^2−^ anion, it was observed that the analytical parameters also showed good linearity between 35.04 and 147.78 μM (Figure 2f), with a LOD and LOQ of 10.51 and 35.04 μM, respectively. Finally, analytical parameters were also evaluated for the determination of the HSO_4_^−^ anion from CdTe-CYA QDs, obtaining a linear range between 6.51 and 123.46 μM (Figure 2h), with LOD and LOQ equal to 1.95 and 6.51 μM, respectively. Increasing further the anion concentrations, a plateau in the emission intensity was observed at 196 and 172 μM for SO_4_^2−^ and HSO_4_^−^, respectively. Furthermore, the statistical analysis indicated that the respective regression models are statistically significant, providing statistical evidence to confirm the relationship between the variables of analyte concentration and emission intensity at a 95% confidence level (Table S1).

ANOVA and the F-test for the statistical significance of the regression (F value) were the criteria used to assess the analytical performance of the proposed models (Table S1). The F value was obtained by dividing the regression mean squares (MSs) by the residual mean squares for each target anion. Thus, the calculated F values were approximately 357.2 (CO_3_^2−^), 1031.3 (HCO_3_^−^), 747.3 (SO_4_^2−^), and 979.1 (HSO_4_^−^), which were much higher than the F-critical values at a significance level of 5%, namely, 10.13 (CO_3_^2−^), 5.59 (HCO_3_^−^), 7.71 (SO_4_^2−^), and 10.13 (HSO_4_^−^).

The performance detection parameter values for CdTe-CYA QDs concerning the target anions are summarized in Table 1, along with examples from the literature.

The few examples found in the literature, for fluorometric sensors for the target anions, present lower LOD values compared to those determined in this study. However, in the literature, the detection studies of these anions do not typically provide a specific value for the LOQ. Regarding the estimated LOD value, it is worth noting that the LOQ values for SO_4_^2−^ and HSO_4_^−^ anions showed values below the maximum levels allowed by the United States Environmental Protection Agency [43] and the World Health Organization (WHO) [44], which are 500 mg·L^−1^ and 250 mg·L^−1^, respectively, whereas there is no established standard for CO_3_^2−^ and HCO_3_^−^ set by regulatory agencies. Thus, the values determined in this study for the quantification of the target anions are well below the established maximum concentrations allowed. Furthermore, some of these reported fluorescent probes are based on organic molecules that require a laborious synthetic procedure. This underscores the intricacies involved in their preparation and the importance of considering practicality alongside analytical sensitivity when selecting suitable detection methods for real-world applications.

### 3.3. Analysis of the QDs’ Emission Profile after the Addition of the Anions

**Figure 3:**
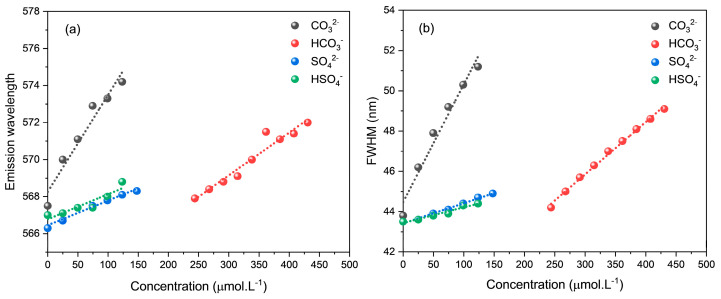
Variation of the emission band spectral position (**a**) and the respective FWHM (**b**) for CdTe-CYA as a function of increasing concentration of the anions (λ_exc_ = 405 nm).

As observed, the fluorescence intensity of CdTe-CYA QDs increased considerably with increasing anion concentration. Simultaneously, with each addition, a slight redshift (<7 nm) and broadening of the emission band (<8 nm) were observed (Figure 3, Table S2). This behavior was more expressive for CO_3_^2−^, followed by HCO_3_^−^, and for SO_4_^2−^ and HSO_4_^−^, there were no significant changes in both the spectral position and the FWHM of the emission band.

Furthermore, it was observed that the variation in FWHM values occurred between 0.1 and 7.3 nm, considering the four studied anions, with CO_3_^2−^ and HCO_3_^−^ being the most expressive. However, it is emphasized that a slight redshift (<7 nm) along with a slight broadening of the emission band (<8 nm) only indicates that changes are occurring on the surface of the QD due to interactions with the anions, while the QD core remains unchanged [45].

### 3.4. Selectivity of the Nanoprobe for Target Anions

**Figure 4:**
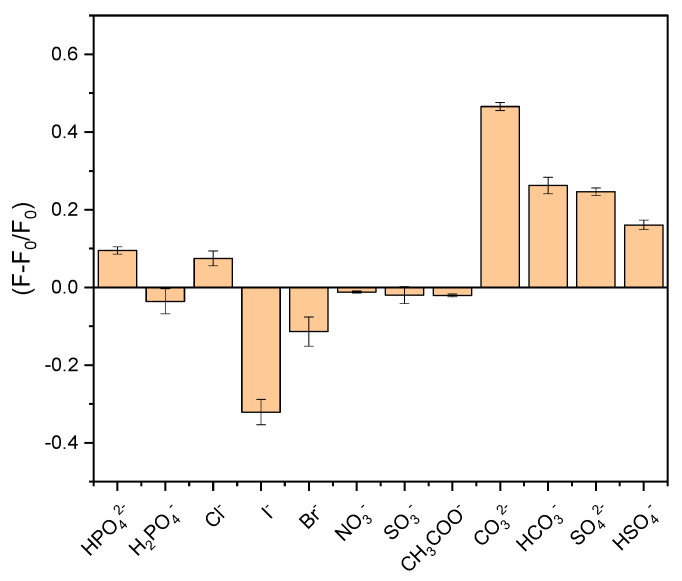
Fluorescence intensity of CdTe-CYA QDs (λ_exc_ = 405 nm) in presence of different anions: HPO_4_^2−^, H_2_PO^4−^, Cl^−^, I^−^, Br^−^, NO_3_^−^, SO_3_^−^, CH_3_COO^−^, CO_3_^2−^, HCO_3_^−^, SO_3_^2−^, HSO_4_^−^.

The sensor’s selectivity toward the anions CO_3_^2−^, HCO_3_^−^, SO_4_^2−^, and HSO_4_^−^ was evaluated by adding other anions at the same concentration. Figure 4 illustrates the change in the fluorescence (F − F_0_)/F_0_ ratio of the nanosensor in response to different tested anions. It is evident that the sensor’s fluorescence exhibited a much more pronounced increase for CO_3_^2−^, followed in this order by HCO_3_^−^, SO_4_^2−^, and HSO_4_^−^. On the other hand, HPO_4_^2−^ and Cl^−^ also showed a more discreet increase in the fluorescence intensity. Additionally, I^−^ and Br^−^, in contrast, led to a decrease in fluorescence intensity, with I^−^ causing a more significant reduction, indicating that this QD could also be a sensor for these halide ions. The remaining tested anions did not induce significant changes in the fluorescence intensity of the nanosensor.

### 3.5. Detection Mechanism of CdTe-CYA QDs

Surface ligands play a crucial role in the interaction between QDs and analytes, strongly affecting the detection mechanisms [46,47]. The stabilizing ligands typically used in aqueous synthesis have a thiol group at one end, which remains attached to the nanoparticle’s surface, and another terminal group such as carboxylic (-COOH) or amino (-NH_2_) ones, which can impart different electrical charges to the QDs depending on the pH. Amino and carboxylic groups can have positive and negative charges, respectively, allowing for electrostatic phenomena to regulate the interaction between the QD surface ligand and anions, in addition to interactions due to hydrogen bonding or van der Waals forces [19,47].

Quantum dots stabilized with CYA may present a strong positive surface charge due to the presence of amino groups that are protonated at pH values around 6 [32]. Thus, it is expected that the CdTe-CYA QDs show electrostatic attraction to anions based on their surface charge, resulting in a change in the spectral profile of the nanoprobes.

There are few cases described in the literature regarding the mechanisms of photoluminescence enhancement in sensors based on QDs [48,49,50], with the quenching mechanism being much more widely observed. Nevertheless, it is known that these interaction mechanisms depend on a series of factors, such as the reactive species involved, electrostatic interaction between the analyte and surface ligands, electron transfer from the QDs’ conduction band to the molecular orbitals of the analyte, and adsorption of the analyte on the nanoparticle surface, among others [51]. In some cases, these interactions can enable an efficient excitonic electron–hole recombination by reducing potential trap density, which are intermediate energy levels between the energy bands of the QD, which are a consequence of both intrinsic and surface defects, thereby increasing radiative recombination and leading to enhanced photoluminescence [52].

Therefore, the detection mechanism between CdTe-CYA QDs and the target anions should be mainly regulated by electrostatic interactions, between the positively charged amino groups anchored on the QDs’ surface and the respective anion. The -NH_3_^+^ group can act as an electron-withdrawing group through an inductive effect, potentially weakening the thiol–QD bond and altering the charge density of the nanoparticle, consequently affecting the emission of the QDs. With the interaction with the anions, the positive charge of the stabilizer is compensated by the negative charge of the analyte, reducing its electron-withdrawing character, favoring more efficient radiative decay processes, and consequently enhancing the emission intensity. Alternatively, as a consequence of this withdrawing character, there may be a change in the flow of charges in the nanoparticle, which, in turn, reduces the charge density in the conduction band, affecting the emission intensity by decreasing the number of possible radiative decays.

## 4. Conclusions

In summary, we prepared and employed CdTe-CYA QDs as analytical nanoplatforms for anion detection (CO_3_^2−^, HCO_3_^−^, SO_4_^2−^, and HSO_4_^−^ ions). The QDs exhibited good linearity, likely due to interactions between the positively charged amino groups of the CdTe-CYA and the respective anion, indicating that the probable detection mechanism is by electrostatic attraction. Analytical parameters, including linear range, LOD, and LOQ, were determined for each CdTe-CYA/anion system, and the obtained values are within acceptable limits. Notably, for SO_4_^2−^ and HSO_4_^−^, the values are below the levels permitted by environmental guidelines. The assessment of analytical performance through an analysis of variance (ANOVA) confirmed the statistical significance (95% confidence level) of the proposed models for detecting CO_3_^2−^, HCO_3_^−^, SO_4_^2−^, and HSO_4_^−^ ions using CdTe-CYA QDs. The selectivity study indicated that the proposed sensor, despite its nonspecific nature, exhibits selectivity to some extent toward the target ions, notably for CO_3_^2−^, for which it showed the best analytical response. Therefore, based on the obtained results, CdTe-CYA QDs can be considered promising nanosensors for the detection of target anions in aqueous media.

## Figures and Tables

**Table 1:** Comparison of various fluorescent probes for the determination of target anions.

Anion	Sensor	Linear Range (μM)	LOD (μM)	LOQ (μM)	Reference
CO_3_^2−^	Eu/CDs@UiO-66-(COOH)_2_	0–350	1.08	-	[36]
	CaF-Tb^3+^	20–100	0.99	-	[37]
	Ureia derivative-CdSe	0.1–100	0.023	-	[38]
	CdTe-CYA QDs	43.0–123.5	12.9	43.0	This work
HCO^3−^	CaF-Tb^3+^	20–100	2.15	-	[37]
	Triazole-naphthalene	2.5–32.5	1.8	-	[39]
	CdTe-CYA QDs	107.3–430.6	32.2	107.3	This work
SO_4_^2−^	Guanidine dyes	2.5–10	0.10	-	[40]
	Bis(diamidocarbazole)	-	1.0	-	[41]
	CdTe-CYA QDs	35.0–147.8	10.5	35.0	This work
HSO_4_^−^	ZnO QDs-benzimidazole	-	0.0032	-	[24]
	Quinazoline-based Co^3+^ complex	0.32–12.5	0.32	-	[42]
	CdTe-CYA QDs	6.5–123.5	2.0	6.5	This work

## Data Availability

Data are contained within the article or Supplementary Materials.

## Supplementary Materials

The following supporting information can be downloaded at: https://www.mdpi.com/article/10.3390/mi15030373/s1, Figure S1: Absorption spectra of CdTe-CYA QDs in the presence of different anion concentrations: (a) CO_3_^2−^, (b) HCO_3_^−^, (c) SO_4_^2−^, and (d) HSO_4_^−^. Table S1: ANOVA and validation of the analytical curve models. Table S2: Results obtained for the interaction of CdTe-CYA and the target anions.
