# Supplementary material for: Development of Fluorescent Sensors for Biorelevant Anions in Aqueous Media Using Positively Charged Quantum Dots
Source: Micromachines (Basel). 2024 Mar 9;15(3):373. doi: 10.3390/mi15030373 (PMC10972362; doi:10.3390/mi15030373)
Supplement: Supplementary file 1 [file micromachines-15-00373-s001.zip › micromachines-2868371-supplementary.pdf]

Supplementary Materials

Development of Fluorescent Sensors for Biorelevant Anions in Aqueous Media Using Positively Charged Quantum Dots

Hitalo J. B. Silva [1], Claudete F. Pereira [1], Goreti Pereira [1,2,*] and Giovannia A. L. Pereira [1,*]

[1] Departamento de Química Fundamental, Universidade Federal de Pernambuco, Recife 50740-560, PE, Brazil; hitalo.silva@ufpe.br (H.J.B.S.); claudete.fernandes@ufpe.br (C.F.P.)
[2] Departamento de Química & CESAM, Universidade de Aveiro, 3810-193 Aveiro, Portugal
* Correspondence: goreti.pereira@ua.pt (G.P.); giovannia.pereira@ufpe.br (G.A.L.P.)

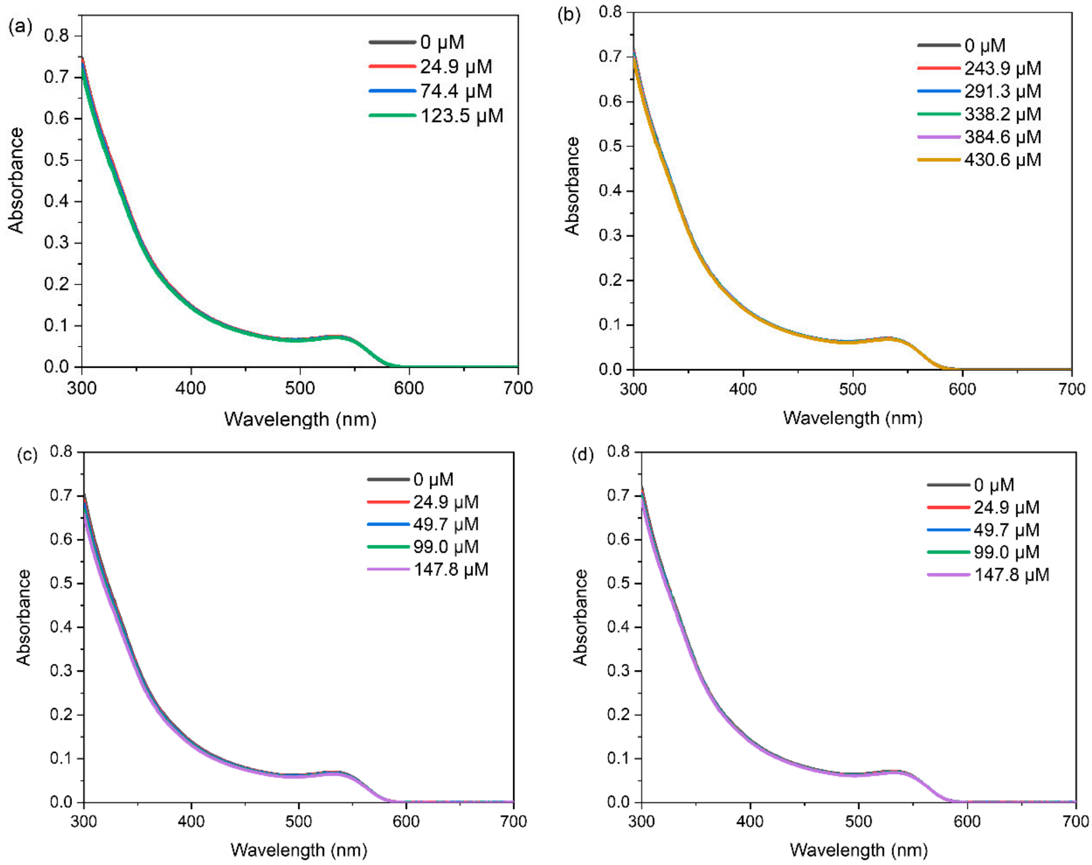

Figure S1. Absorption spectra of CdTe-CYA QDs in the presence of different anion concentrations: (a) CO_3^{2-} (b) HCO_3^- (c) SO_4^{2-}, and (d) HSO_4^-.

Table S1. ANOVA and validation of the analytical curve models.

Anion	Source of variation	DF	SS	MS	F value	F-critical	P-value
CO_3^{2-}	Regression	1	0.200736	0.200736	357.19	10.13	0.000323
	Residual	3	0.001686	0.000562			
HCO_3^-	Regression	1	0.174477	0.174477	1031.26	5.59	$7.342x10^{-9}$
	Residual	7	0.001184	0.000169			
SO_4^{2-}	Regression	1	0.041889	0.041889	747.35	7.71	$1.065x10^{-5}$
	Residual	4	0.000224	$5.605x10^{-5}$			
HSO_4^-	Regression	1	0.0286684	0.028668	979.11	10.13	$7.172x10^{-5}$
	Residual	3	$8.784x10^{-5}$	$2.928x10^{-5}$			

(DF) Degrees of Freedom; (SS) Sum of Squares; (MS) Mean Squares.

Table S2. Results obtained for the interaction of CdTe-CYA and the target anions.

Anion	Concentration (μM)	λ_{emi} (nm)	FWHM (nm)	ΔFWHM (nm)	pH
CO_3^{2-}	0	567.5	43.8	-	5.8
	24.9	570.0	46.2	2.4	-
	49.8	571.1	47.9	4.0	-
	74.4	572.9	49.2	5.4	-
	99.0	573.3	50.3	6.4	-
	123.5	574.2	51.2	7.3	6.0
HCO_3^-	0	567.2	43.7	-	5.8
	243.9	567.9	44.2	0.6	-
	267.6	568.4	45.0	1.3	-
	291.3	568.8	45.7	2.0	-
	314.8	569.1	46.3	2.7	-
	338.2	570.0	47.0	3.3	-
	361.5	571.5	47.5	3.9	-
	384.6	571.1	48.1	4.4	-
	407.7	571.4	48.6	4.9	-
	430.6	572.0	49.1	5.4	6.2
SO_4^{2-}	0	566.3	43.5	-	5.8
	24.9	566.7	43.6	0.1	-
	49.8	567.4	43.9	0.4	-
	74.4	567.5	44.1	0.6	-
	99.0	567.8	44.4	0.9	-
	123.5	568.1	44.7	1.2	-
	147.8	568.3	44.9	1.4	6.4
HSO_4^-	0	567.0	43.5	-	5.8
	24.9	567.1	43.6	0.1	-
	49.8	567.4	43.8	0.3	-
	74.4	567.4	43.9	0.5	-
	99.0	568.0	44.3	0.8	-
	123.5	568.8	44.4	0.9	6.0
